# Supplementary material for: Refining Boolean models with the partial most permissive scheme
Source: Bioinformatics. 2025 Mar 22;41(4):btaf123. doi: 10.1093/bioinformatics/btaf123 (PMC12021794; doi:10.1093/bioinformatics/btaf123)
Supplement: btaf123_Supplementary_Data [file btaf123_supplementary_data.zip › supplementary data.pdf]

Supplementary data

Nadine Ben Boina[1,2], Brigitte Mossé[1], Anaïs Baudot[2,3,4], and Elisabeth Remy[1]

[1] *I2M, CNRS, Aix Marseille University, Marseille, France.*
[2] *Aix Marseille Univ, INSERM, MMG, Marseille, France*
[3] *CNRS, Marseille, France*
[4] *Barcelona Supercomputing Centre, Barcelona, Spain*

04 - 2023

S1 Construction of Multivalued Refinements of a BM

Given a BM f on $\mathbb{B}^n$, we recall that given $j_0 \in \{1, \ldots, n\}$, the map f_{j_0} can be set in a disjunctive normal form (DNF). In other words, $f_{j_0}(x)$ is written as a disjunction of clauses that are themselves conjunctions of radicals $w\, x_j$ as below:

$$f_{j_0}(x) = \bigvee_{h \in \{1,\ldots,s_{j_0}\}} \; \bigwedge_{k \in \{1,\ldots,r_{j_0,h}\}} w_{j_0,h,k}\, x_{j_{j_0,h,k}} \quad , \tag{1}$$

where operators $\wedge$, $\vee$ stand for AND, OR respectively, and the $w_{j_0,h,k}$ are equal to ε (empty string), or $\neg$ (NON).
Moreover, this DNF is supposed to be a shortest one - this ensures that components involved in the expression of f_{j_0} are effective regulators of g_{j_0}, and avoids redundancy.

A way to build a refinement h of f on $X = \prod_{j=1}^{n} \{0, 1, \ldots, m_j\}$ is then the following:

- Starting from (1), and the current variable becoming $x \in X$, for each $j = j_{j_0,h,k}$ in (1) for which $m_j > 1$, we choose a partition of $\{0, \ldots, m_j\}$ of the form $\{0, \ldots, s\} \cup \{s+1, \ldots, m_j\}$ and we replace $w_{j_0,h,k}\, x_{j_{j_0,h,k}}$ with $x_{j_{j_0,h,k}} \geq s+1$ if $w_{j_0,h,k} = \varepsilon$, and with $x_{j_{j_0,h,k}} < s+1$ if $w_{j_0,h,k} = \neg$. Clauses with indices j such that $m_j = 1$ remain unchanged.
- In this way, we obtain a function of the variable $x \in X$ with values in $\mathbb{B}$; let $\mathcal{H}_{j_0}$ be defined by $\mathcal{H}_{j_0}(x) = +1$ when this value is 1, and $\mathcal{H}_{j_0}(x) = -1$ when it is 0.
- Finally, we define the refinement $h = (h_1 \ldots, h_n)$ setting for $j \in \{1, \ldots, n\}$

$$h_j(x) = max(0, min(m_j, x_j + \mathcal{H}_j(x))),$$

that ensures that the map h is actually a map from X to itself.

S2 Comparison of Reachability Properties in a BM and Multi-valued Refinements

Proposition 1. *Let f be a BM on $\mathbb{B}^n$, the map h a refinement of f on $X = \prod_{j=1}^{n} \{0, 1, \ldots, m_j\}$, and $J = \{j \in \{1, \ldots, n\} \; ; \; m_j > 1\}$. We suppose that no component g_j of J is self-inhibited.*

Set a and a' two Boolean states of $\{0,1\}^n$, and b and b' the elements of X obtained replacing the coordinate a_j of a (resp. a'_j of a') by m_j when $a_j = 1$ (resp. $a'_j = 1$), for each $j \in J$. If there exists a trajectory from a to a' in the asynchronous STG of f, then there exists a trajectory from b to b' in the asynchronous STG of h.

Proof. It is sufficient to prove this property in the case where there is a transition $a \to a'$ in the asynchronous STG of f, and $a_1 \neq a'_1$. Suppose for instance that $a_1 = 0$ and $a'_1 = 1$ (the case $a_1 = 1$ and $a'_1 = 0$ can be treated in a similar way). Then the states b and b' differ only by their first coordinates, $b_1 = 0$ and $b'_1 = m_1$.

In the previous notation, a conjunctive clause of the form $\bigwedge_{k \in \{1,\ldots,r\}} w_k \, x_{j_k}$ in the logical formula of f_1 is made true by a. This implies that the corresponding clause of $\tilde{h}$ built as above is made true by b.

- In the case $m_1 = 1$, this gives a transition from b to b'.

- In the case $m_1 > 1$, there is a transition from b to the state $b^{(1)}$ obtained from b by changing b_1 into 1. Considering the hypothesis that x_1 does not occur in the clause $\bigwedge_{k \in \{1,\ldots,r\}} w_k \, x_{j_k}$, this can be iterated until getting all the transitions $b \to b^{(1)} \to \cdots \to b^{(m_1)} = b'$, as expected. □

Remark 1. *If a component g_j of J is self-inhibited, this proposition is irrevocably defeated. This can be illustrated for instance setting $n = 2$, and for $x \in \mathbb{B}^2$,*
$f_1(x) = \neg x_1 \;\vee\; \neg x_2 \,, \quad f_2(x) = \neg x_1 .$

- An example of refinement h of f on $X = \{0,1,2\} \times \{0,1\}$ is given by
$\mathcal{H}_1(x) = +1 \textit{ iff } x_1 < 2 \;\vee\; \neg x_2 \,, \quad \mathcal{H}_2(x) = +1 \textit{ iff } x_1 < 1 \,.$
There is a transition from 11 to 01 in the asynchronous STG of f, but no trajectory from 21 to 01 in the asynchronous STG of h.

- Another example of refinement h' of f on $X = \{0,1,2\} \times \{0,1\}$ is given by
$\mathcal{H}'_1(x) = +1 \textit{ iff } x_1 < 1 \;\vee\; \neg x_2 \,, \quad \mathcal{H}'_2(x) = +1 \textit{ iff } x_1 < 2 \,.$
The state 10 is a fixed point of f, and the lonely attractor of its asynchronous dynamics. There is a trajectory from 01 to 10 in the asynchronous STG of f, but no trajectory from 01 to 20 in the asynchronous STG of h'. Moreover, there is a new attractor in this STG of h', that is a cycle of length 2 between 01 and 11.

x	$f(x)$
0 0	1 1
0 1	1 1
1 0	1 0
1 1	0 0

x	$h(x)$
0 0	1 1
1 0	2 0
2 0	2 0
0 1	1 1
1 1	2 0
2 1	1 0

x	$h'(x)$
0 0	1 1
1 0	2 1
2 0	2 0
0 1	1 1
1 1	0 1
2 1	1 0

We are especially interested in the fixed points of the dynamics, for which the way we build refinements gives the following.

Proposition 2. *Let f be a BM on $\{0,1\}^n$, the map h a refinement of f on $X = \prod_{j=1}^{n} \{0, 1, \dots, m_j\}$, and $J = \{j \in \{1, \dots, n\} \; ; \; m_j > 1\}$. We suppose that no component g_j of J is self-inhibited.*

- *Set ω be a fixed point of f, and ω' the element of X obtained replacing the coordinates ω_j of ω equal to 1 by m_j, for each $j \in J$. Then ω' is a fixed point of h, and all the fixed points of h are obtained in this way.*
- *Let suppose that the attractors of the asynchronous dynamics of f are l fixed points $\omega^{(1)}$, ..., $\omega^{(l)}$. Then the l fixed points $\omega'^{(1)}$, ..., $\omega'^{(l)}$ of h obtained as above are the lonely attractors of the asynchronous dynamics of h.*

Proof.

- The fact that ω' is a fixed point of h comes from the inequalities required to be a refinement and from $\alpha(\omega') = \{\omega\}$.

  Conversely, suppose that a state $x \in X$ is a fixed point of h. Then, by the hypothesis, all the coordinates x_j with $j \in J$ have to be equal to 0 or m_j, and $\alpha(x)$ is reduced to one Boolean state y. Finally, the state x being a fixed point of h, the state y is necessarily a fixed point of f, as expected.

- Set $x \in X$. By the hypothesis, there is a trajectory from x to a state z whose coordinates z_j with $j \in J$ are all equal to 0 or m_j. Then, $\alpha(z)$ is reduced to one Boolean state y. There is a trajectory from y to some fixed point $\omega^{(k)}$ of f. By Proposition 1, there is thus a trajectory from z to $\omega'^{(k)}$. In conclusion, all the states x of X lead to a fixed point of h, that achieves the proof.

□

S3 Generalization of the Completeness Property of the m.p. Scheme to the Partial m.p. Schemes

For the sake of convenience, in this supplementary, trajectories from a state x to a state y will be denoted in a contracted manner $x \underset{---}{\overset{f}{\longrightarrow}} {}^* \; y$.

Completeness property of the m.p. scheme

We begin recalling Paulevé & al theorem ([9]), and give a detailed constructive proof.

Let us consider an integer $n \geq 1$, a BM f of dimension n, and a refinement h of f defined on $X = \prod_{j=1}^{n} \{0, 1, \ldots, m_j\}$, where $m_j \in \mathbb{N}^*$ for each $j \in \{1, \ldots, n\}$, and at least one of the m_j is > 1.

For $j \in \{1, \ldots, n\}$, we denote by F_j the map defined by $F_j(x) = f_j(x) - x_j$ for $x \in \mathbb{B}^n$, and by H_j the map defined by $H_j(x) = h_j(x) - x_j$ for $x \in X$ (these maps are with values in $\{-1, 0, +1\}$).

Let x be an element of X. Let us call *m.p. state compatible with* x any element $\hat{x}$ of $X_{m.p.} = \{0, 1, i, d\}^n$ such that for each $j \in \{1, \ldots, n\}$,

- if $x_j = 0$, then $\hat{x}_j = 0$,
- if $x_j = m_j$, then $\hat{x}_j = 1$,
- if $x_j \notin \{0, m_j\}$, then $\hat{x}_j = i$ or d.

Remark 2. *In both definitions of the m.p. scheme related to f and of the multivalued refinements of f, the associated components are supposed to assume that any intermediate level of some component g (the levels i and d, the levels l such that $0 < l < m_j$ for some j) can be considered as levels where g is, or is not, active.*

For $x \in X$, the set
$\alpha(x) = \{x' \in \mathbb{B}^n \,;\, \forall j \in \{1, \ldots, n\}, \, (x_j = 0 \Rightarrow x'_j = 0) \, and \, (x_j = m_j \Rightarrow x'_j = 1)\}$,
and for $x \in X_{m.p.}$ the set
$\gamma(x) = \{x' \in \mathbb{B}^n \,;\, \forall j \in \{1, \ldots, n\}, \, (x_j = 0 \Rightarrow x'_j = 0) \, and \, (x_j = 1 \Rightarrow x'_j = 1)\}$
are introduced to this end.

Hence, the proof of the following theorem is essentially based on the fact that if $x \in X$, and $\hat{x}$ is an element of $X_{m.p.}$ compatible with x, then $\alpha(x) = \gamma(\hat{x})$.

Theorem 1. *In the previous notation, let $x \xrightarrow[asyn]{h} {}^* \, y$ be a trajectory in the asynchronous STG of h. For any element $\hat{x}$ of $X_{m.p.}$ compatible with x, there exists in the STG of the m.p. dynamics of f a trajectory $\hat{x} \xrightarrow[m.p.]{f} {}^* \, \hat{y}$ such that $\hat{y}$ is compatible with y.*

Proof. It is clear that it is sufficient to prove the result in the case where the asynchronous trajectory is reduced to one transition, that is $x \xrightarrow[asyn]{h} y$.

Let $\hat{x}$ be an element of $X_{m.p.}$ compatible with x, and $j \in \{1, \ldots, n\}$ the integer such that $x_j \neq y_j$.

For convenience, we suppose that $j = 1$, and we detail the construction of $\hat{x} \xrightarrow[m.p.]{f} {}^* \, \hat{y}$ in the case $H_1(x) > 0$, the case $H_1(x) < 0$ being similar.

- If $m_1 = 1$, $x_1 = \hat{x}_1 = 0$ and $y_1 = 1$, then the existence of $x' \in \beta(x) = \gamma(\hat{x})$ such that $F_1(x') > 0$ gives
$$\hat{x} = (0, \hat{x}_2, \ldots, \hat{x}_n) \;\rightarrow\; (i, \hat{x}_2, \ldots, \hat{x}_n) \;\rightarrow\; (1, \hat{x}_2, \ldots, \hat{x}_n) \,.$$
- If $m_1 > 1$, $x_1 = \hat{x}_1 = 0$ and $y_1 = 1$, then the existence of $x' \in \beta(x) = \gamma(\hat{x})$ such that $F_1(x') > 0$ gives
$$\hat{x} = (0, \hat{x}_2, \ldots, \hat{x}_n) \;\rightarrow\; (i, \hat{x}_2, \ldots, \hat{x}_n) \,.$$

- If $m_1 > 1$, $x_1 = l$, where $0 < l < m_1 - 1$ and $y_1 = l + 1$, then $\hat{x}_1 = i$ or d and $\hat{x}$ is compatible with y :

$$\text{stay on } \hat{x}.$$

- If $m_1 > 1$, $x_1 = m_1 - 1$, $y_1 = m_1$ and $\hat{x}_1 = i$, then the existence of $x' \in \beta(x) = \gamma(\hat{x})$ such that $F_1(x') > 0$ gives
$$\hat{x} = (i, \hat{x}_2, \ldots, \hat{x}_n) \ \rightarrow \ (1, \hat{x}_2, \ldots, \hat{x}_n) \ .$$

- If $m_1 > 1$, $x_1 = m_1 - 1$, $y_1 = m_1$ and $\hat{x}_1 = d$, then the existence of $x' \in \beta(x) = \gamma(\hat{x})$ such that $F_1(x') > 0$ gives
$$\hat{x} = (d, \hat{x}_2, \ldots, \hat{x}_n) \ \rightarrow \ (i, \hat{x}_2, \ldots, \hat{x}_n) \ \rightarrow \ (1, \hat{x}_2, \ldots, \hat{x}_n) \ .$$

□

Completeness property of the partial m.p. schemes

We consider now an integer $n \geq 1$, a BM f of dimension n, a non-empty subset J of $\{1, \ldots, n\}$, and a refinement h of f defined on $X = \prod_{j=1}^{n} \{0, 1, \ldots, m_j\}$, where $m_j \in \mathbb{N}^*$ for each $j \in \{1, \ldots, n\}$, and $m_j > 1$ if and only if $j \in J$.

As above, for $j \in \{1, \ldots, n\}$, we denote by F_j the map defined by $F_j(x) = f_j(x) - x_j$ for $x \in \mathbb{B}^n$, and by H_j the map defined by $H_j(x) = h_j(x) - x_j$ for $x \in X$.

Let x be an element of X. We call *$^J m.p.$ state compatible with x* any element $\hat{x}$ of $X_{^J m.p.}$ such that for each $j \in \{1, \ldots, n\}$,

- if $x_j = 0$, then $\hat{x}_j = 0$,
- if $x_j = m_j$, then $\hat{x}_j = 1$,
- if $x_j \notin \{0, m_j\}$, then $\hat{x}_j = i$ or d.

Theorem 2. *In the previous notation, let $x \xrightarrow[asyn]{h} {}^* \, y$ be a trajectory in the asynchronous STG of h. For any element $\hat{x}$ of $X_{^J m.p.}$ compatible with x, there exists in the STG of the $^J m.p.$ dynamics of f a trajectory $\hat{x} \xrightarrow[^J m.p.]{f} {}^* \, \hat{y}$ such that $\hat{y}$ is compatible with y.*

Proof. The proof is an easy adaptation of the one of Theorem 1: we restrict ourselves to the case where the considered trajectory is reduced to one transition $x \xrightarrow[asyn]{h} y$, with $x_1 \neq y_1$ and $H_1(x) > 0$. Let $\hat{x}$ be an element of $X_{^J m.p.}$ compatible with x. A suitable trajectory $\hat{x} \xrightarrow[^J m.p.]{f} {}^* \, \hat{y}$ is obtained on the following way:

- If $1 \in J$ the construction of $\hat{x} \xrightarrow[^J m.p.]{f} {}^* \, \hat{y}$ is the same than in the proof of Theorem 1.

- If $1 \notin J$, then $x_1 = 0$, $y_1 = 1$ and the existence of $x' \in \beta(x) = \gamma(\hat{x})$ such that $F_1(x') > 0$ gives

$$\hat{x} = (0, \hat{x}_2, \ldots, \hat{x}_n) \ \rightarrow \ (1, \hat{x}_2, \ldots, \hat{x}_n) \ .$$

□

S4 Multivalued refinement of the BM of Early Hematopoietic Stem Cell Aging

You can find the model in ".bnet" format within the GitHub repository's "Examples/Hérault, Léonard et al (2022)" directory. The entirety of the logical rules for the multivalued refinement presented in the results section is as follows:

$Egr1 = Gata2 \,\&\, Junb$
$Junb = Egr1 : 2 \,|\, Myc$
$Blacf1 = Myc$
$Myc = Cebpa \,\&\, Blacf1$
$Fli1 : 2 = Junb \,|\, Gata1 : 2 \,\&\, !Klf1)$
$Gata2 : 2 = (Gata2 \,\&\, !Gata1 \,\&\, !Zfpm1) \,|\, (Egr1 \,\&\, !Gata1 \,\&\, !Zfpm1 \,\&\, !Spi1)$
$Spi1 : 2 = (Spi1 \,\&\, !Gata1) \,|\, (Cebpa \,\&\, !Gata1 \,\&\, !Gata2 : 2)$
$Cebpa = (Gata2 \,\&\, !Ikzf1) \,|\, (Spi1 \,\&\, !Ikzf1)$
$Gata1 : 2 = Fli1 \,|\, (Gata2 \,\&\, !Spi1) \,|\, (Gata1 \,\&\, !Ikzf1 \,\&\, !Spi1)$
$Klf1 = Gata1 \,\&\, !Fli1 : 2$
$Tal1 = Gata1 \,\&\, !Spi1$
$Ikzf1 = Gata2$
$Zfpm1 = Gata1$
$CDK46CycD = Bclaf1 \,|\, Myc$
$CIPKIP = Junb$

To simplify notation, we denote the activity of a node by its label (e.g., $Gata2$, not x_{Gata2}). Additionally, for multivalued components, when the threshold of regulation is other than 1, it is denoted by ":level". For instance, $Gata2 : 2$ signifies that $x_{Gata2} = 2$ whereas $Gata2$ means $x_{Gata2} = 1$.

S5 Multivalued refinement of the BM of Asymmetric Stem Cell Division in *Arabidopsis Thaliana* Root

You can find the multivalued refinement discussed in the corresponding result section within the GitHub repository's "Examples/García-Gómez, Mónica L et al (2020)" directory.
